# Recurrent Cardioembolic Strokes Due to Marantic Endocarditis As the Initial Presentation of Colorectal Adenocarcinoma

**DOI:** 10.7759/cureus.24183

**Published:** 2022-04-16

**Authors:** Denis Babici, Phillip M Johansen, Adrian Rodriguez-Hernandez, Shad Sommerville, Brian Snelling, Timothy D Miller

**Affiliations:** 1 Neurology, Florida Atlantic University Charles E. Schmidt College of Medicine, Boca Raton, USA; 2 Neurological Surgery, Boca Raton Regional Hospital, Boca Raton, USA

**Keywords:** brain tumors cns tumors, brain thrombectomy, mri images, colorectal cancer, cranial nerve paralysis

## Abstract

Metastases to the brain from primary colorectal carcinoma are rare. Existing literature describing cranial nerve palsy from metastatic colorectal cancer is scattered. To our knowledge, we are the first to describe the combination of CN deficits V, VII, and XII as the initial presentation of colorectal malignancy. The authors present the case of a patient with no past medical history who presented with multiple cranial nerve deficits of the right trigeminal, facial, and hypoglossal nerves. MRI of the brain revealed a mass in Meckel's cave, which explained the involvement of the trigeminal nerve (CN V) but not the facial (CN VII) and hypoglossal (CN XII) nerves. Further workup revealed multiple cardioembolic strokes caused by nonbacterial thrombotic endocarditis (NBTE). Extensive workup for the cause of his NBTE and subsequent cerebrovascular events revealed colorectal adenocarcinoma.

## Introduction

The relationship between thromboembolism and cancer was first described by Trousseau in 1865. Trousseau and colleagues reported a 15% incidence of clinically relevant thromboembolic events in patients with cancer and up to 50% in post-mortem studies [1]. Stroke mechanisms are often unconventional or ambiguous in those with underlying malignancy. Compared to a 30% cryptogenic rate in the general population, nearly 50% of cancer-associated strokes are deemed cryptogenic after evaluation. A common hypothesis is that many cryptogenic strokes in patients with cancer are from cardioembolic manifestations of cancer-mediated hypercoagulability such as nonbacterial thrombotic endocarditis (NBTE), which consists of sterile, platelet-fibrin vegetations on cardiac valves [2]. Additionally, central nervous system (CNS) involvement occurs in approximately 30% of patients with cancer [3]. Of these patients, cerebrovascular accidents, either ischemic or hemorrhagic, are the second leading cause of CNS involvement. The patient may present with single or multiple cranial nerve palsies, which can be sequential, discrete, unilateral, bilateral, painful, or painless. Additionally, the presentation can be acute, subacute, or recurrent. Syndromes causing multiple adjacent cranial neuropathies, such as Vernet, Schmidt, Collet-Sicard, Jackson, Tapia, and Villaret, have been well described. However, non-adjacent cranial neuropathies are rare and difficult to attribute to a structural lesion [3].

## Case presentation

A 48-year-old man recently diagnosed with Bell’s palsy by his primary care physician presented to the emergency department with a four-week history of back pain and dysarthria, alongside right-sided facial numbness, dysgeusia, lagophthalmos, and blurred vision. He also reported intermittent nausea, a 20-lb unintentional weight loss, and intermittent subjective fevers. Upon examination, the patient had right-sided impairment of facial muscle function, including drooping of the brow and corner of the mouth, flattening of the nasolabial fold, and impaired closure of the eye and mouth. Protrusion of the tongue displayed right-sided deviation. His gag reflex, along with all other cranial nerves, was intact. Motor, sensory, and cerebellar examinations were unremarkable.

**Figure 1:**
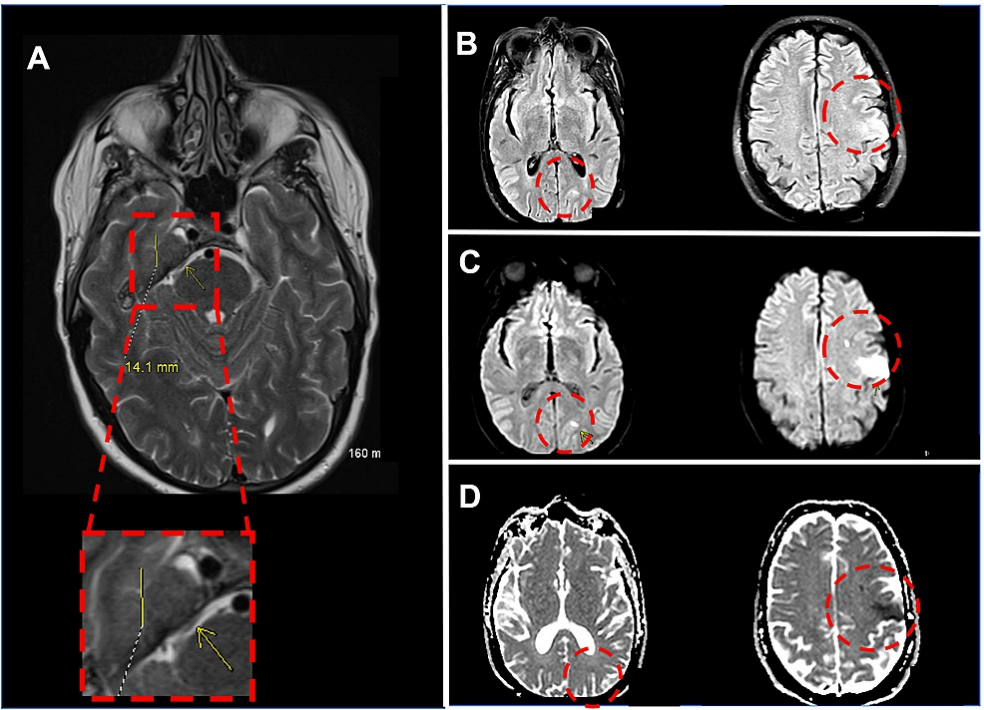
MRI of the brain A: 1.4 cm mass at the level of the posterior margin of Meckel's cave. B: Flair showing multiple, left-sided, subacute cardioembolic strokes. C: Diffusion-weighted imaging (DWI) showing multiple, left-sided, subacute cardioembolic strokes. D: Antibody-drug conjugate (ADC) showing multiple, left-sided, subacute cardioembolic strokes.

Computed tomography (CT) scan without contrast, CT angiography, and CT perfusion of the head revealed no abnormalities. Magnetic resonance imaging (MRI), however, showed a 1.4 cm mass at the level of the right posterior margin of Meckel's cave (Figure 1A). Multiple small, scattered lesions were also found in the right frontal and occipital lobes and bilateral cerebellum. The lesions showed characteristics of different aging and appeared embolic in origin (Figures 1B, 1C, 1D).

**Figure 2:**
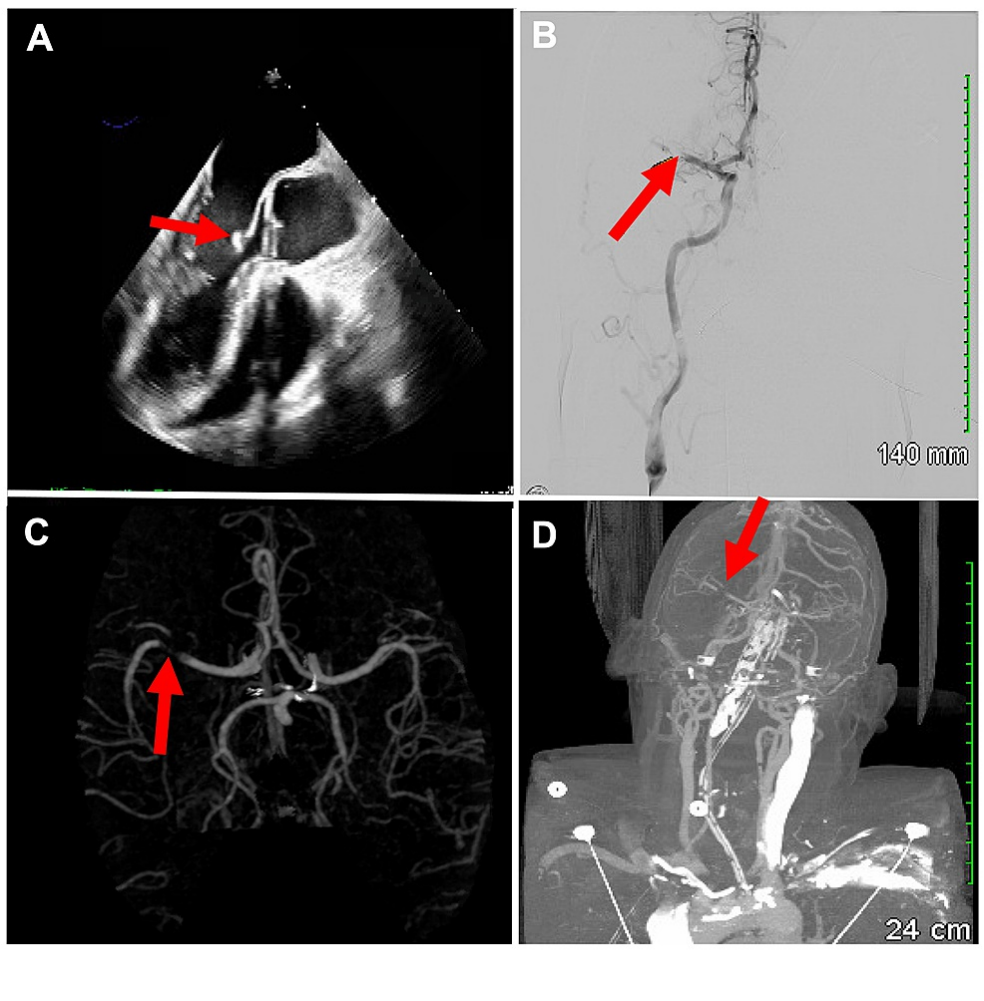
Acute left MCA occlusion due to marantic endocarditis A: Transesophageal echocardiogram showing a mass on the atrial side of the anterior leaflet of the mitral valve. B. Cerebral angiogram showing right M1 MCA occlusion. C & D: Computed tomography angiography (CTA) of the brain showing right M1 MCA occlusion.

MRI of the lumbar spine showed diffuse marrow replacement with multiple focal lesions throughout, and the patient was started on aspirin, clopidogrel, and high-intensity atorvastatin. A strong suspicion of the underlying metastatic process in the setting of his imaging findings and recent weight loss warranted further workup. A transesophageal echocardiogram showed a mass of approximately 1.2 x 0.5 cm on the atrial side of the anterior leaflet at the mitral valve (Figure 2A). The valvular vegetation led to suspicion of bacterial endocarditis, but subsequent blood cultures were negative; thus, nonbacterial thrombotic endocarditis (NBTE) was considered due to his concomitant hypercoagulable state.

On hospital day two, the patient suddenly developed generalized left-sided weakness, a 30-second staring episode, emesis, and an ensuing change in mental status. On evaluation, the patient was hypoxic and subsequently placed on a non-rebreather. Urgent head and neck CT angiogram with perfusion revealed occlusion of the right middle cerebral artery (MCA) (Figures 2C, 2D), and the patient was taken for emergent thrombectomy (Figure 2B).

The thrombectomy tissue was thought to represent soft tissue emboli, but unfortunately, a detailed pathological examination was not performed. Due to the suspected relationship between his hypercoagulable state and ischemic stroke, the team opted for bone marrow biopsy, which revealed poorly differentiated metastatic adenocarcinoma likely secondary to colorectal cancer. Gastroenterology was consulted and discovered the primary mass via colonoscopy. The lesion was biopsied and diagnosed as poorly differentiated colonic adenocarcinoma. 

## Discussion

The evaluation of patients presenting with cranial nerve palsies is challenging as the differential diagnosis is broad. The presentation may be due to singular or multiple cranial nerve palsies, which can be sequential, discrete, unilateral, bilateral, painless, or painful. Skull base syndromes causing combinations of adjacent cranial neuropathies, such as Vernet, Schmidt, Collet-Sicard, Jackson, Tapia, and Villaret syndromes, have been well described [3]. However, non-adjacent cranial neuropathies, which are considerably rarer, are difficult to attribute to a structural lesion because of the distance between cranial nerve nuclei. Additionally, the presentation could be acute, subacute, chronic, or recurrent. 

Regardless of the presentation, anatomic localization is the first step in evaluation. Possible locations of cranial nerve lesions include the brain stem, meninges, or base of the skull. Additionally, lesions may be extracranial, resulting in accompanied systemic symptoms [3]. The largest case series of multiple cranial neuropathies were reported by Keane [3], who found that tumors were the cause of 30% of multi-cranial nerve neuropathies, followed by vascular disease (12%), trauma (12%), infection (10%), Guillain-Barré syndrome (6%), idiopathic cavernous sinusitis (5%), surgery (5%), multiple sclerosis and autoimmune demyelinating encephalomyelitis (5%), Fisher syndrome (3%), functional disease (3%), diabetes mellitus (2%), and other benign, miscellaneous causes. Non-localized neuropathies comprised 18% of cases. Cranial nerve VI is most commonly affected, followed by VII, V, III, and X, respectively [4]. The most common combination of cranial nerves is the III and VI nerves, V and VI nerves, and V and VII nerves [4]. The specific combination of cranial nerve deficits seen in the previously described case, namely V, VII, and XII, has not been described in the existing literature. This patient’s symptoms should most likely be attributed to leptomeningeal disease that wasn’t present on initial cerebrospinal fluid (CSF) cytology specimens. Alternatively, multiple cardioembolic strokes are another potential etiology, especially in a patient with colorectal cancer.

There are myriad factors that may contribute to thromboembolism in cancer patients. For instance, the interaction between tumor cells and both monocytes and macrophage lineages results in the release of various pro-inflammatory cytokines, including tumor necrosis factors, interleukin-1, and interleukin-6, which damage the endothelial lining and thus convert it into a thrombogenic surface. Furthermore, the interaction between malignant cells and macrophages can activate platelets, factor XII, and factor X, thereby inducing clotting on the already hypercoagulable vascular lining. In addition, tumor cells release thrombogenic substances, including cysteine protease and tissue factor, which can activate factor X and factor VII to further stimulate the thrombogenic cascade [5]. 

Along with the effects on the clotting pathway, the immobility and immunosuppressed state secondary to chemotherapy, radiotherapy, and other aggressive pharmacologic treatments often administered via intravenous access with central line placement can predispose patients with cancer to neurologic infection, which can also incite thromboembolic events [5]. Among cancer-associated thromboembolism, venous thromboembolic events are most common. Other reported manifestations include arterial thrombosis, disseminated intravascular coagulation (DIC), NBTE, migratory superficial thrombophlebitis, and thrombotic microangiopathy [1]. One study revealed that cerebrovascular events, both ischemic and hemorrhagic, are the second leading cause of CNS involvement in patients with cancer following metastases. Nevertheless, cerebral ischemic stroke, as the initial manifestation of previously undetected cancer, is extremely rare (0.4%) [3]. 

NBTE, as described in the presenting case, is the most frequent cause of cerebral ischemia in patients with cancer, regardless of whether or not the stroke is the initial manifestation of cancer [6]. Lastly, factors to be considered with the diagnosis of NBTE should include the distribution of occluded vessels and identification of thrombus, which can be detected via criterion standard transesophageal echocardiography when evaluating the left atrial appendage, and exclusion of other possible causes such as infective endocarditis [1,7].

## Conclusions

The most important issue in diagnosing cranial nerve palsy is whether it matches an expected pattern and whether it is "isolated." While it is conceivable for multiple cranial nerve palsies to have a microvascular cause, all patients with more than a single nerve palsy or with other neurologic findings must have an extensive workup (neuro-logic examination and MRI) before the diagnosis is confirmed. NBTE must be considered for patients with a background of malignancy and new onset of ischemic cerebral stroke. The diagnosis should depend on the distribution of occluded vessels, identification of thrombus, and exclusion of other possible causes. Healthcare providers must work collaboratively in these complex cases. The approach to managing NBTE should be directed at preventing systemic embolization with long-term anticoagulation and treating the underlying malignancy.
